# Seminal Plasma Exposures Strengthen Vaccine Responses in the Female Reproductive Tract Mucosae

**DOI:** 10.3389/fimmu.2019.00430

**Published:** 2019-03-12

**Authors:** Romain Marlin, Marie-Thérèse Nugeyre, Nicolas Tchitchek, Matteo Parenti, Cécile Lefebvre, Hakim Hocini, Fahd Benjelloun, Claude Cannou, Silvia Nozza, Nathalie Dereuddre-Bosquet, Yves Levy, Françoise Barré-Sinoussi, Gabriella Scarlatti, Roger Le Grand, Elisabeth Menu

**Affiliations:** ^1^IDMIT Department, U1184 ≪ Immunology of Viral Infections and Autoimmune Diseases ≫ (IMVA), CEA, IBFJ, Université Paris-Sud, Inserm, Fontenay-Aux-Roses, France; ^2^MISTIC Group, Department of Virology, Institut Pasteur, Paris, France; ^3^Vaccine Research Institute - VRI, Hôpital Henri Mondor, Créteil, France; ^4^Équipe 16 Physiopathologie et Immunothérapies dans l'Infection VIH, Institut Mondor de Recherche Biomédicale - INSERM U955, Créteil, France; ^5^Infectious Diseases Department, San Raffaele Scientific Institute, Milan, Italy; ^6^Groupe Henri-Mondor Albert-Chenevier, Service d'Immunologie Clinique, Assistance Publique-Hôpitaux de Paris (AP-HP), Créteil, France; ^7^International Division, Institut Pasteur, Paris, France; ^8^Viral Evolution and Transmission Unit, San Raffaele Scientific Institute, Milan, Italy

**Keywords:** female reproductive tract (FRT), seminal plasma, vaccine, mucosa, HIV-1

## Abstract

HIV-1 sexual transmission occurs mainly via mucosal semen exposures. In the female reproductive tract (FRT), seminal plasma (SP) induces physiological modifications, including inflammation. An effective HIV-1 vaccine should elicit mucosal immunity, however, modifications of vaccine responses by the local environment remain to be characterized. Using a modified vaccinia virus Ankara (MVA) as a vaccine model, we characterized the impact of HIV-1^+^ SP intravaginal exposure on the local immune responses of non-human primates. Multiple HIV-1^+^ SP exposures did not impact the anti-MVA antibody responses. However, SP exposures revealed an anti-MVA responses mediated by CD4^+^ T cells, which was not observed in the control group. Furthermore, the frequency and the quality of specific anti-MVA CD8^+^ T cell responses increased in the FRT exposed to SP. Multi-parameter approaches clearly identified the cervix as the most impacted compartment in the FRT. SP exposures induced a local cell recruitment of antigen presenting cells, especially CD11c^+^ cells, and CD8^+^ T cell recruitment in the FRT draining lymph nodes. CD11c^+^ cell recruitment was associated with upregulation of inflammation-related gene expression after SP exposures in the cervix. We thus highlight the fact that physiological conditions, such as SP exposures, should be taken into consideration to test and to improve vaccine efficacy against HIV-1 and other sexually transmitted infections.

## Introduction

Semen is a complex fluid composed of a cellular fraction containing spermatozoa and leukocytes, and a non-cellular fraction, the so-called seminal plasma (SP) including diverse set of components such as cytokines, chemokines, fibrils, immunoglobulins, complement factors, and bacteria (1–3). Semen deposition in the female reproductive tract (FRT) during intercourse induces physiological modifications (4), such as the induction of pro-inflammatory cytokine/chemokine production (5, 6), and upregulation of inflammatory upstream regulator expression, such as Cyclooxygenase-2 (COX-2) (7), and leukocyte infiltration (8, 9). These modifications of the local environment have been described to promote fertility and are not due to intercourse itself, but require SP exposure (10), as described *in vitro* and *in vivo* in mice (5) and pigs (11) as well as humans (10). SP exposure also induces the recruitment of regulatory T cells to the FRT, showing that SP does not have only a pro-inflammatory effect (12).

Semen is the main vector of HIV-1 transmission. The virus is present as both cell-free particles in the SP and infected mononuclear cells (13). Both have been shown to be infectious in animal models (14–16) and human cervical explants (17). SP is not a passive carrier of viral particles (18); it contributes to reduce the protective acidic pH of the vagina (19) and favors the attachment of virions to target cells (20). In contrast, several antiviral factors have been characterized in SP (21). The composition of semen varies according to the presence and stage of HIV-1 infection. In particular, the cytokine and chemokine network is different between the SP from HIV-1^neg^ and HIV-1^+^ individuals (22), pro-inflammatory factors are higher in the SP of HIV-1^+^ individuals (23). The effect of HIV-1^+^ SP on the local environment of the FRT is still uncharacterized.

The FRT mucosae are the main portal of HIV-1 entry during heterosexual intercourse. As a consequence, the induction of efficient mucosal immune responses in the FRT is a strategy to prevent heterosexual transmission of HIV-1. However, many preclinical efficacy studies in animal models and non-human primates (NHP) in particular, testing vaccine candidates or microbicides, used a cell-free culture medium-derived virus inoculum for the challenge phase, which does not allow evaluation of the physiological effects of SP on local immunity. We have recently shown that systemic immunization with an MVA vector-based vaccine was able to induce vaccine-specific CD8^+^ T cells in all FRT compartments in macaques (24). However, the effect of SP on such vaccine-induced mucosal immune responses is completely unknown.

The aim of the present study was to determine the effect of vaginal HIV-1^+^ SP exposure on local immunity and vaccine-specific responses in the FRT. We used an MVA vector-based vaccine as a vaccine model and show that SP exposures increase the specific CD8^+^ T-cell response, myeloid dendritic cells (mDC) recruitment and inflammation-related gene expression. A local specific CD4^+^ T-cell response was also detected after vaginal SP exposures. Multi-parameter approaches clearly identified the cervix as the most affected compartment in the FRT.

## Materials and Methods

### Constitution of the SP Pool

Human semen was collected from 14 HIV-1 infected subjects (among them, 13 were naive for antiretroviral therapy) attending the Infectious Disease Dept., OSR, under the supervision of Dr. Sivia Nozza. Patients were informed of the study and signed an informed consent (HIVSPERM study, protocol number 5/INT/2014 of 2014/02/06). At collection, the median age was 37.5 years (min 28; max 55), the median infection time was 5.5 years (min 2; max 20), the median plasma viral load was 2,468 copies/ml (min 45; max 119,116) and their median CD4 count was 636.5 (min 371; max 10,027). SP was obtained after liquefaction of the semen at 37°C for 30 min and centrifugation at 1,000 × g for 10 min. Supernatant were collected, pooled and stored at −80°C.

### Cytokine/Chemokine Quantification

Pro-inflammatory and anti-inflammatory cytokines/chemokines and TGFβ isoforms were measured in SP pool by Luminex assays (cytokine human magnetic 25-plex panel; Invitrogen, Courtaboeuf, France and TGFβ 1,2,3 Magnetic Bead Kit, MerckMillipore, Germany). HIV-1 RNA viral load was determined in SP pool by COBAS TaqMan HIV-1 test V2.0 (Roche).

### Experimental Design

On day zero (D0) and D58, the macaques received two subcutaneous injections per time point in the right and left side of the upper back, delivering 2 × 1 ml of inoculum containing a total of 4 × 10^8^ plaque-forming units (PFU) of recombinant MVA-HIV-1 expressing the Gag, Pol, and Nef proteins from HIV-1 strain LAI (ANRS-MVA HIV-B, MVATG17401, Transgene Ltd., France). The animals were monitored daily for signs of disease, appetite loss and lethargy. A physical examination was performed at each blood sampling and each inoculation. All experimental procedures (handling, immunization, blood sampling) were conducted after sedation with ketamine hydrochloride (Rhône-Mérieux, Lyon, France, 10 mg/kg). An intramuscular injection of a synthetic variant of progesterone (Depoprovera, 30 mg, Pfeizer, France) was given 42 days after the first vaccine injection to synchronize their hormonal cycle. On D70, D72, D74, and D76, animals were exposed intra-vaginally to 800 μl 1X PBS (control group *n* = 6) or 800 μl SP-pool/PBS (v:v) (SP group, *n* = 6). The animals were sedated 77 days post-vaccination with ketamine hydrochloride (10 mg/kg) and euthanized by intravenous injection of 180 mg/kg sodium pentobarbital.

### Sample Collection and Cell Isolation

Blood, serum and vaginal fluid were collected before and after each vaccine inoculation and at the time of euthanasia. Collection of serum, cervicovaginal fluids, and PBMC was previously described (24), briefly serum was isolated by centrifugation, cervicovaginal fluid was collected with a Weck-Cel Spear and PMBCs were isolated in heparin CPT tubes. LNs and tissues were collected at necropsy. LN cells were obtained by mechanical dissociation. FRT tissues (vagina and cervix which included both ectocervix and endocervix, uterus, and fallopian tubes) were isolated and cut into small pieces. Each tissue was digested for 1 h at 37°C with agitation in digestion buffer, consisting of RPMI 1640 (Fisher Scientific, Illkirch, France), collagenase IV (0.3 mg/ml, Sigma Aldrich, St Quantin Fallavier, France), fetal calf serum (5%, Fisher Scientific), HEPES (0.025 M, Fisher Scientific), DNase (0.1 mg/ml, Roche, Mannheim, Germany), and antibiotics (Fisher Scientific). Undigested pieces were subjected to up to three more digestion steps. Cell suspensions from LNs and FRT tissues were filtered through 70-μm sterile nylon cell strainers (BD Biosciences).

### Immune Phenotyping

Whole blood, LN cells and cells from FRT compartments were analyzed by flow cytometry. The cells were incubated with the antibodies listed in Table S1, washed, and then fixed with FACS lysing buffer or BD Cell Fix solution. A Fortessa 2-UV 6-Violet 2-Blue 5-Yelgr 3-Red laser configuration was used (BD Biosciences), with Diva (BD) and FlowJo 9.8.3 (Tristar, USA) software. At least 500 events for rare cell populations (i.e., pDC) were recorded.

### Cellular Responses

Specific cellular immune responses were evaluated with *in vitro* stimulation assays. The cells were incubated for 5 h at 37°C in DMEM medium (Fisher Scientific) supplemented with 10% FCS and antibiotics, alone or medium with 0.3 PFU/cell of live wild-type MVA, or PMA (5 ng/ml) and ionomycin (500 ng/ml) (Sigma Aldrich). Brefeldin A was then added (5 μg/ml, Sigma Aldrich) and the cells incubated for a further 10 h at 37°C. For HIV-1 stimulation, cells were incubated with 4 μg/ml overlapping Gag peptide pools (HIV-1 LAI strain) in DMEM medium supplemented with 10% FCS, antibiotics and costimulatory antibodies, for 1 h at 37°C, then for an additional 4 h with brefeldin A (5 μg/ml). The cells were stained with LIVE/DEAD® Fixable Blue Dead Cell Stain (Thermo Fisher) to assess viability, fixed, and then permeabilized with BD Fix&Perm reagent (BD Bioscience). The antibodies listed in Table S2 were used for intracellular staining. At least 5,000 events in the CD8^+^ T cell gate were recorded. The gating strategy was as described elsewhere (25). Briefly, cytokine and activation marker expression was evaluated in CD4^+^ and CD8^+^ T cells, and Boolean gate analyses were performed with FlowJo software. The percentages of cells positive for cytokines and activation markers between unstimulated and MVA-stimulated cells were then compared.

### Antibody Responses

Specific antibodies were measured by EIA in sera and vaginal fluids, as described previously (24). First, 96-well MaxiSorp microplates (Nunc, Thermo Fisher) were coated overnight with 10^5^ PFU/well wtMVA (Transgene, Illkirch, France) in NaHCO_3_/Na_2_CO_3_ buffer, or 1 μg/ml p24 antigen (kind gift from Bernard Verrier, LBTI UMR5305) in PBS. The plates were then blocked for 1 h with PBS containing 3% (w/v) bovine serum albumin (BSA, Sigma Aldrich) or PBS containing 10% skimmed milk. The plates were washed five times with PBS containing 0.1% Tween 20 and 10 mM EDTA, then incubated with two-fold serial dilutions of macaque fluids diluted in PBS containing 1% BSA for 1 h at room temperature (to detect anti-MVA IgG/IgA) or PBS containing 1% skimmed milk and 0.05% Tween 20 for 1 h at 37°C (to detect anti-HIV IgG), starting at 1:50 for serum and 1:20 for vaginal fluid. The plates were then washed five times and incubated for 1 h with a 1:20,000 dilution of horseradish peroxidase-conjugated goat anti-monkey H+L chain IgG (Bio-Rad, Marne-la-Coquette, France) or a 1:5,000 dilution of horseradish peroxidase-conjugated goat anti-monkey IgA (Alpha Diagnostic international, San Antonio, TX). The plates were washed five times, then 100 μL o-phenylenediamine dihydrochloride (OPD) (Sigma Aldrich) was added and the plates incubated for 30 min at room temperature in the dark. The reaction was stopped by adding 2N H_2_SO_4_. Absorbance was measured at 492 nm with a spectrophotometer (Tecan, Lyon, France), and the data analyzed using Magellan software (Tecan). Antibody titers were calculated by extrapolation from the OD as a function of a serum dilution curve and defined as the dilution of the test serum reaching 2 OD of the corresponding preimmune serum or vaginal fluid, tested at 1:50 and 1:30, respectively.

### RNA Extraction and Hybridization

Tissue biopsies were immediately immersed in RLT-beta-mercaptoethanol 1/100 lysis buffer (Qiagen, Courtaboeuf, France), disrupted, and then homogenized with a TissueLyser LT (Qiagen). RNA was purified using Qiagen RNeasy microkits. Contaminating DNA was removed using the RNA Cleanup step of the RNeasy microkit. Purified RNA was quantified with a ND-8000 spectrophotometer (NanoDrop Technologies, Fisher Scientific, Illkirch, France) before being checked for integrity on a 2100 BioAnalyzer (Agilent Technologies, Massy, France). cDNA was synthesized and biotin-labeled using the Ambion Illumina TotaPrep RNA amplification kit (Applied Biosystem/Ambion, Saint-Aubin, France). Labeled cRNA was hybridized on Illumina Human HT-12V4 BeadChips, which target 47,323 probes corresponding to 34 694 genes. The manufacturers' protocols were followed.

### Data Analysis

Comparison between the control group and the SP-exposed group (antibody and T cell responses, and cell population abundance) were analyzed using the two-tailed Mann-Whitney test. The two-tailed Wilcoxon signed rank test was used to compare paired conditions (T cell responses within each animal group). Polyfunctional profiles of MVA-specific CD8^+^ T cells were compared between the two groups using the Chi-squared test. The statistical threshold of significance was fixed at *p* < 0.05 for these tests.

Microarray were analyzed using R/Bioconductor software. Gene expression values were quantile normalized. Differentially expressed genes were identified with a paired non-parametric *t*-test (*q*-value < 0.05), based on a 1.2-fold-change cutoff. Functional enrichment analysis was performed using QIAGEN's Ingenuity Pathway Analysis (IPA, QIAGEN, Redwood City, https://www.qiagenbioinformatics.com/products/ingenuity-pathway-analysis/). Hierarchical clustering presented in the heatmaps were generated with the Euclidian metric and complete linkage methods. The correlation between molecular and cellular data were computed using Spearman coefficients of correlation (*R* > 0.70 and *p* < 0.01), based on the abundance of cell populations and normalized gene expression values across differentially expressed genes in the vagina, cervix, and uterus. Significant correlations were restricted to those between cell population abundances and gene expression levels. Microarray raw data are available from the EBI-ArrayExpress database under accession number E-MTAB-7639.

## Results

### The Humoral Response Is Not Modified by SP Exposures

Two groups of six cynomolgus macaques immunized by subcutaneous injection of 4 × 10^8^ PFU of rMVA-HIV-1 at weeks 0 and 8, were exposed to PBS, as controls, or to a SP pool at days 70, 72, 74, and 76 following first vaccine injection. The seminal plasma pool was obtained from HIV-1 infected patients and contained 22,000 vRNA copies/ml, and inflammatory as well as anti-inflammatory cytokines/chemokines (Table 1). As expected, TGFβ was the most abundant.

**Table 1:** Cytokine and chemokine composition of the SP pool.

**Soluble factors**	**Concentration (pg/ml)**
TGF-β1	112,967
TGF-β2	16,892
TGF-β3	56,766
IL-6	29
IL-7	33
IL-15	139
IL-1RA	350
IL-8	4,330
CCL11 (Eotaxin)	11
CCL4	57
CCL5	108
CCL2	837
CXCL9	1,036
CXCL10	2,156

All animals had been treated with synthetic progesterone on day 42 in order to ensure a similar cervico-vaginal mucosa structural organization and immune status of all animals at time of intravaginal exposure. In addition, progesterone treatment, mimicking luteal phase or contraceptive medication in women, has been shown to increase susceptibility to SIV transmission in macaques (26), therefore representing a “worse case” scenario for exploring local vaccine induced immunity.

One day (D77 of the study) following the last SP/PBS exposure, animals were euthanized and immune responses explored in tissues collected at necropsy. The MVA-specific IgG titers in the sera of control and SP-exposed animals were similar (30,307 ± 6,884 and 31,315 ± 8,390, respectively; mean ± SEM of six animals) (Figure S1A). In addition, we detected MVA-specific IgA in the sera of both, control (1,314 ± 328), and SP-exposed animals (1,955 ± 1,162) without any statistical difference (Figure S1B). In the vaginal fluids, the MVA-specific IgG and IgA titers of the control group (389 ± 111 and 151 ± 54, respectively) were not significantly different from those of the SP-exposed group (219 ± 52 and 205 ± 62, respectively) (Figures S1D,E). The MVA we used also encodes HIV-1 Gag, Pol and Nef proteins, as a consequence, animals may raise anti-HIV responses which may be boosted by exposure to SP obtained from HIV-1 infected donors. However, anti-HIV Gag IgG titers in the SP-exposed group (847 ± 714) were not significantly different from those of the control group (155 ± 54) (Figure S1C). HIV Gag-specific IgG were detected in sera but not in vaginal fluids (Figure S1F). Overall, these results suggest that SP exposures do not significantly modify the vaccine-specific humoral responses.

### SP Exposures Revealed a Local Specific CD4^+^ T-Cell Response

**Figure 1:**
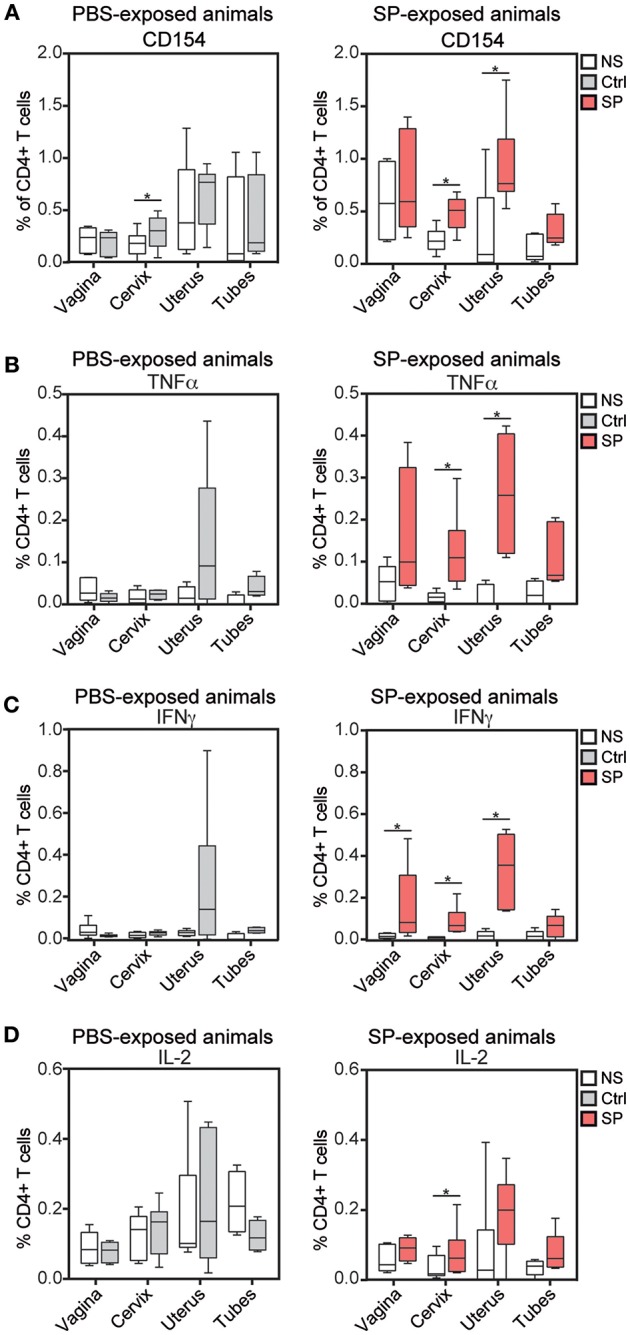
SP exposure enhances vaccine-specific mucosal CD4^+^ T cell responses. Percentage of CD154^+^ **(A)**, TNF-α^+^ **(B)**, IFN-γ^+^ **(C)**, and IL-2^+^ **(D)** cells among CD4^+^ T cells after *in vitro* stimulation with medium (white) or wt MVA (gray for control animals, left panel and red for SP exposed animals, right panel) in FRT tissues. The Wilcoxon signed rank test (*) was used to compare NS vs. wt MVA stimulation antigens (*p < 0.05).

We evaluated the MVA-specific CD4^+^ T cell response in the blood, lymph nodes (LNs) and FRT following *ex-vivo* re-stimulation of isolated leukocytes. HIV-Gag-specific CD4^+^ T-cell responses were very weak and mainly detected in the blood. No differences between the two groups were detected (data not shown). MVA-specific CD4^+^ T-cells were mainly found in the blood and axillary LNs draining the vaccine injection site (Figure S2A), as previously described (24). No differences between the two groups were detected in these compartments. Within the FRT of the control animals, expression of CD154, used as a marker of activated cells, was significantly increased only in CD4^+^ T cells of the cervix (from 0.06 to 0.50% of total CD4^+^ T cells) following stimulation with MVA and relative to unstimulated cells (Figure 1A, left panel). Also not statistically significant, MVA-specific CD4^+^ T cells producing TNF-α or IFN-γ were found in the uterus of three of six control animals (Figures 1B,C, left panel). In contrast, MVA-specific CD4^+^ T-cells from the cervix and the uterus of all animals exposed to SP showed significantly increased CD154 expression (from 0.23 to 0.69%) (Figure 1A, right panel) associated with significantly increased production of TNF-α (from 0.03 to 0.30%) (Figure 1B, right panel), IFN-γ (from 0.04 to 0.22%) (Figure 1C, right panel), and IL-2 (from 0.02 to 0.21%) (Figure 1D, right panel). Percentage of IFN-γ producing CD4^+^ T cells was also increased in vagina of SP exposed animals. Also not statistically significant, unstimulated CD4^+^ T cells in the vagina of SP-exposed animals expressed a higher percentage of CD154 (from 0.21 to 1.00%) (Figure 1A, right panel) than those of control animals (from 0.06 to 0.32%) (Figure 1A, left panel), suggesting an effect of SP exposures on the basal activation state of vaginal CD4^+^ T cells.

Overall, SP exposures appeared to enhance anti-MVA response mediated by CD4^+^ T cells.

### SP Exposures Increased Specific CD8^+^ T Cell Responses in the Cervix

**Figure 2:**
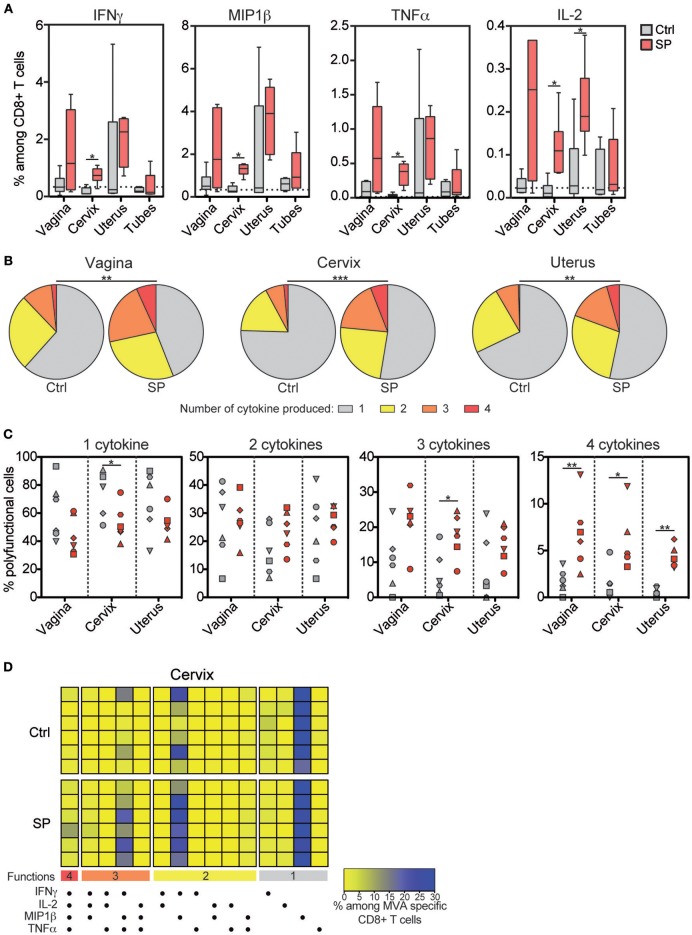
Frequency and quality of vaccine-specific mucosal CD8^+^ T cell responses increase after SP exposures. **(A)** Percentage of IFN-γ^+^, MIP-1β ^+^, TNF-α^+^, and IL-2^+^ cells among MVA specific CD8^+^ T cells in FRT tissues after *in vitro* stimulation. Control animals (Ctrl) are indicated in gray and SP exposed animals (SP) in red. The horizontal dotted line indicates the mean background signal measured in unstimulated cells. **(B)** Pie chart graphs indicating the number of cytokines produced by the MVA-specific CD8^+^ T cells in the mucosal compartments. The means of the six animals of each group are represented in the pie charts. **(C)** Number of cytokines produced among the MVA specific CD8^+^ T cells in control (gray) and SP exposed (red) animals. Each symbol represents an animal. **(D)** Polyfunctional CD8^+^ T-cell profiles analyzed by Boolean gating in cervical tissue are represented as a heat map. Each colored horizontal line indicates one animal [*n* = 6 for the control group (Ctrl) and *n* = 6 for the SP exposed group (SP)]. The Mann-Whitney (*) was used to compare animal groups (*p < 0.05) and the Chi-squared test to compare the pie charts (**p < 0.005 and ***p < 0.001).

We monitored the vaccine response mediated by CD8^+^ T cells in the blood, LNs and FRT of the control and SP-exposed animals using *ex vivo* re-stimulation assay. HIV-Gag-specific CD8^+^ T-cell responses were very weak and detected in the blood of few animals. No differences between the two groups were detected (data not shown). MVA-specific CD8^+^ T cell responses in the blood and LNs were detectable, but not different, between the two groups (Figure S2B). By contrast, percentages of IFN-γ (from 0.27 to 1.09% of total CD8^+^ T cells), MIP-1β (from 0.80 to 1.55%), TNF-α (from 0.11 to 0.53%) and IL-2-producing (from 0.06 to 0.24%) CD8^+^ T cells were statistically higher in the cervix of SP exposed animals compared to controls (Figure 2A). The percentage of MVA-specific uterine IL-2^+^ CD8^+^ T cells were also statistically higher in SP-exposed animals (Figure 2A). Higher proportion of polyfunctional MVA specific CD8^+^ T-cells, measured as the number of cytokines produced by a single cell, were detected in vaginal, cervical, and uterine of the SP-exposed animals compared to controls (Figure 2B), including those cells producing all four cytokines (IFN-γ, TNF-α, MIP-1β, and IL-2) (Figure 2C) and three cytokines (Figures 2C,D). Overall, these results provide evidence that SP exposure increase both the strength and the quality of the response against vaccine vector mediated by FRT CD8^+^ T cells.

### SP Exposures Induced Local Immune Cell Recruitment in the FRT

**Figure 3:**
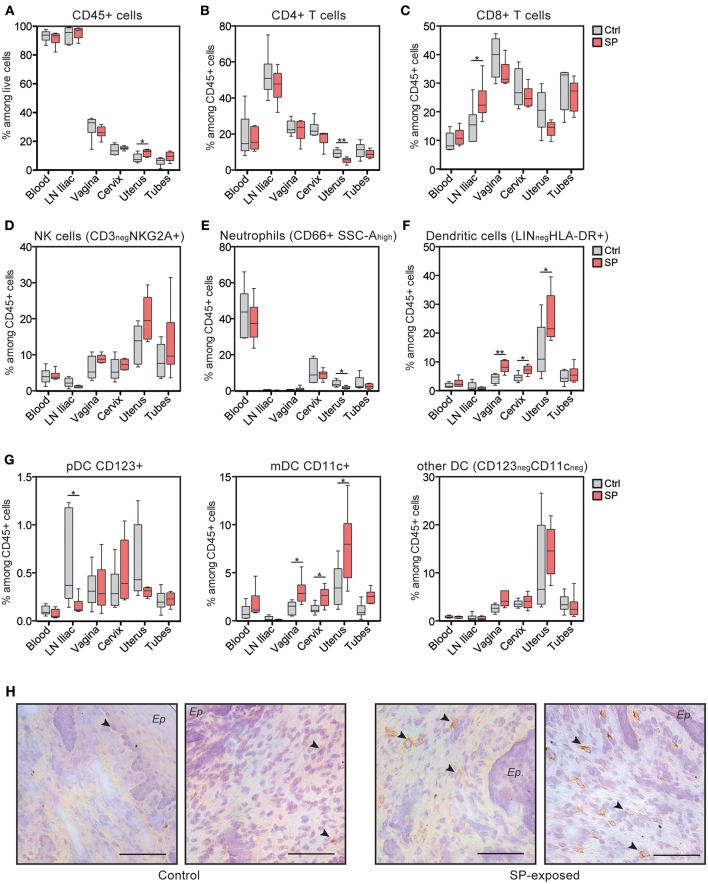
CD11c^+^ mDC are recruited to the FRT of SP exposed animals. **(A)** Distribution of leukocytes among living cells. Percentage of CD4^+^ T cells **(B)**, CD8+ T cells **(C)**, NK cells **(D)**, neutrophils **(E)**, and total dendritic cells **(F)** among leukocytes in the various compartments. **(G)** Percentage of dendritic cell subtypes [i.e., plasmacytoid DC (pDC), mDC, and other DC]. Control animals (Ctrl) are indicated in gray and SP exposed animals (SP) in red. The Mann-Whitney test (*) was used to compare animal groups (*p < 0.05). **(H)** Localization of mDC with anti-CD11c antibody in vaginal tissue sections from control (left panel) and SP-exposed animals (right panel). Black arrows indicate brown CD11c^+^ cells and the black bar indicates 100 μm. Ep, epithelium.

Our results show that SP exposures affect cellular immunity in the FRT. We therefore hypothesize that this could be the result of higher stimulation and/or recruitment of FRT immune cells, including lymphocytes and antigen presenting cells (APC). We thus characterized the immune cell composition in the various FRT compartments of the animals of both groups. The percentage of leukocytes (CD45^+^ cells) and CD4^+^ T cells were not affected by SP, except in the uterus (Figures 3A,B). However, the frequency of CD8^+^ T cells were significantly increased in the iliac LN of the SP-exposed animals (23.7 ± 2.7%; mean ± SEM) compared to controls (15.6 ± 2.7%) (Figure 3C). Analysis of the innate immunity components showed the percentage of NK cells and neutrophils to be similar in both groups (Figures 3D,E), except in the uterus, for which the percentage of neutrophils was significantly lower in the SP-exposed group (1.8 ± 0.3%) than in control group (4.0 ± 0.8%). The frequency of dendritic cells was higher in the vaginal, cervical, and uterine tissues of the SP-exposed group (8.4 ± 0.8, 7.2 ± 0.6, and 25 ± 3.5%, respectively) compared to controls (respectively 4.3 ± 0.6%, 4.6 ± 0.6% and 13.8 ± 3.8%) (Figure 3F). Phenotypic characterization of the dendritic subpopulations showed increased recruitment of mDC (CD11c^+^) after SP exposures (Figure 3G). *In situ* observations showed that CD11c^+^ cells were mainly localized to the subepithelial area (Figure 3H). Altogether, these results show that SP exposures induce the recruitment of lymphoid and myeloid dendritic cells in all FRT compartments.

### SP Exposures Mostly Affects Expression of Immune Related Genes in the Cervix

**Figure 4:**
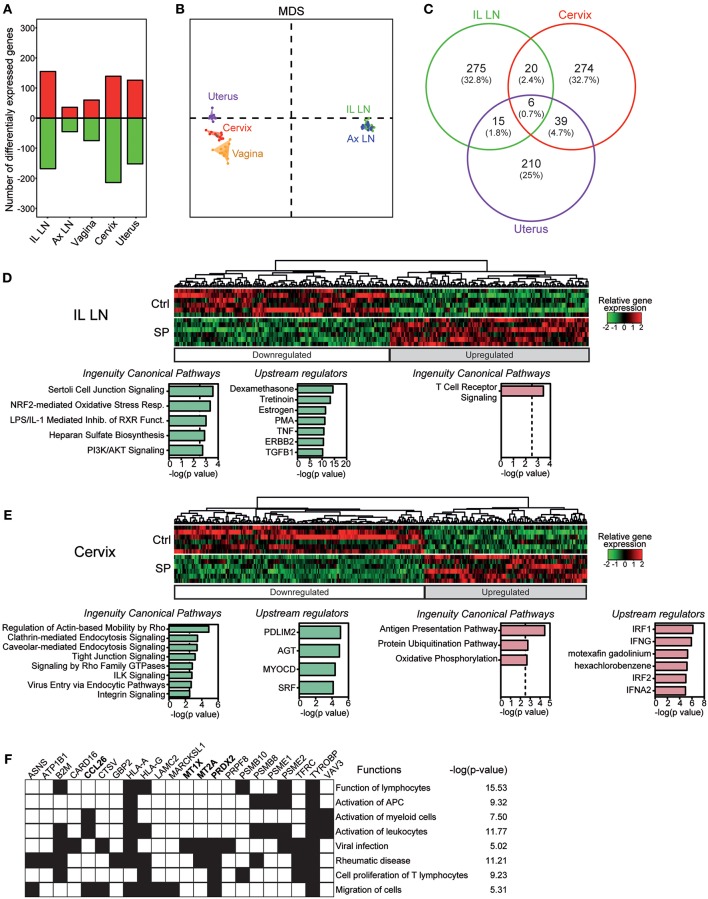
SP exposures impact mainly immune-related gene expression in the cervix. **(A)** Number of down-regulated (green) and upregulated (red) genes affected by SP exposures in the various compartments. **(B)** MDS representation of the whole dataset based on the DEG list for at least one condition. **(C)** Venn diagram showing the overlap between the list of DEG in the iliac LN, cervix and uterus. Heatmaps of relative gene expression in the iliac LN **(D)** and cervix **(E)**. Associated canonical pathways and upstream regulators for down-regulated (green) and up-regulated (red) genes are detailed. **(F)** Biological processes found to be significantly over-represented (*p* < 0.05) in the lists of up-regulated genes targeted by upstream regulators in cervix. The main associated functions are shown.

We compared the transcriptomic profiles of axillary and iliac LNs, vaginal, cervical, and uterine tissues from SP-exposed and control animals. SP exposures modified the expression of 316 genes in the iliac LNs, 134 in the vagina, 80 in the axillary LNs, 339 in the cervix and 270 in the uterus (Figure 4A). Multidimensional scaling (MDS) representation, constructed based on the set of genes found to be differentially expressed (DEG) in a least comparison, showed that the samples from the FRT are segregated and distinct from LNs genes (Figure 4B). We focused in a first step, our analyses on the cervix, uterus and iliac LNs since the number of DEG in the vagina and axillary LNs were lower relative to other tissues. The Venn diagram underscores the specificity of the response of the iliac LNs, cervix, and uterus to SP exposures, as the number of common DEG was low (Figure 4C). The relative expression of DEG in the iliac LNs, cervix, and uterus are represented by a heatmap in Figures 4D,E and Figure S3, respectively. We separately analyzed downregulated and up-regulated gene sets resulting from SP exposures using Ingenuity Pathway Analysis (IPA). Functional enrichment of the most significant canonical pathways and upstream regulators (*p* < 0.003) are detailed under each heatmap. In the iliac LNs, SP exposures downregulated genes associated with stress responses and cell activation (PI3K/AKT signaling, oxidative stress response, TNF, PMA, estrogen, etc.), as well as genes associated with immunomodulation (LPS/IL-1 mediated inhibition of RXR function, tretinoin, TGFB1, dexamethasone, etc.) (Figure 4D). In contrast, up-regulated genes were associated with TCR signaling. The same analyses performed on the cervix showed that SP exposures down-regulated genes associated with cell-cell interactions (ILK, integrin, and tight junction signaling) and those associated with endocytosis and macropinocytosis (clathrin or caveolar endocytosis and Rho-associated pathways) (Figure 4E). In contrast, SP exposures of the cervix upregulated genes associated with antigen presentation (antigen presentation pathway and protein ubiquitination), and upstream regulators involved in immune function and inflammation (IRF1 and IRF2, IFNG, and IFNA2) (Figure 4E). The upstream regulators of these DEG are associated with immune cell functions and activation and inflammatory disease (i.e., rheumatic disease) (Figure 4F). Analyses performed on the uterus showed an effect on upstream regulators associated with the PIK3/AKT pathway, hormones (progesterone), and angiogenic factors (Vascular endothelial growth factor) (Figure S3); however, no significant canonical pathway was found except that of calcium signaling. Similarly, only one pathway related to glycolysis was significantly upregulated in the vagina and found to be statistically over-represented (*p* < 0.0001). Overall, these results show that SP exposures of the FRT mostly affect the expression of genes of the cervix.

### SP Exposures Affect Cervical Inflammatory Responses

**Figure 5:**
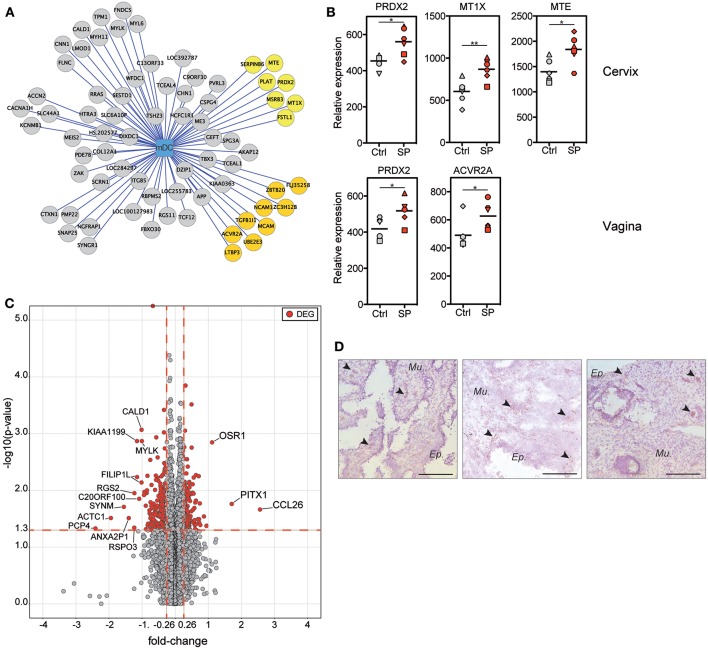
Upregulation of inflammation-related gene expression is associated with mDC recruitment after SP exposures. **(A)** Graph showing the correlations between the mDC abundance and DEG expression in the FRT (vagina, cervix, and uterus). Each node of the graph corresponds to a biological variable and links between the nodes correspond to significant positive correlations (Spearman correlation coefficient). Genes are represented by circles and the mDC cell population by the square. Gene circles colored in yellow are known to be involved in immune-related process. **(B)** Relative expression of inflammation-related genes identified in **(A)** that are upregulated by SP exposures. Each symbol represents one animal from control (gray) or SP group (red). The Mann-Whitney test (*) was used to compare gene expression between the two groups (*p < 0.05). **(C)** Volcano plots showing fold-change (FC, x axis) and statistical significance distribution (–log(*p*-value), y axis) for DEG between to PBS or SP exposure in the cervix. **(D)** Location of CCL26-producing cells in cervical tissue sections from SP exposed animals (*n* = 3). Black arrows indicate brown CCL26^+^ cells and the black bar indicates 100 μm.

We generated a correlation analysis to integrate flow cytometry and transcriptomic data. We restricted the analysis to positive correlations between DEG found in the FRT compartments (vagina, cervix and uterus) and mDCs, which were the cells recruited to the FRT after SP exposures (Figure 5A). Seventy-two genes had an expression profile correlated with the mDC abundance. Among these genes, 16 are known to be involved in immune responses or oxidative stress (Figure 5A in yellow). The expression of some of the same genes increased following SP exposures. For example, the expression of PRDX2 and the metallothioneins (MTE and MT1X), which are involved in protection against oxidative stress (27), positively correlated with the presence of mDCs and increased in cervical tissue following SP exposures (Figure 5B). In addition, these genes are also controlled by upstream regulators associated with cervical upregulated DEG (Figure 4F). The expression of PRDX2 and ACVR2A, a receptor involved in the activin/follistatin pathway (28), also increased in vaginal tissue following SP exposures (Figure 5B).

Our results show that the effects of SP exposures on immunity are mainly localized in cervical tissue. We thus represented the DEG by volcano plots to identify potential biomarkers associated with SP exposures in the cervix (Figure 5C). Three genes were strongly upregulated by SP exposures: OSR-1, PITX-1, and CCL26. The OSR-1 and PITX-1 genes code for transcriptional regulators (29, 30). The CCL26 gene is controlled by upstream regulators associated with cervical upregulated DEG (Figure 4F). CCL26 is a chemokine involved in the regulation of inflammatory processes (31). Immunohistochemistry staining of SP-exposed cervical tissue showed CCL26 producing cells localized both within the epithelium and the mucosa (Figure 5D).

Overall, our transcriptomic analyses provide evidence that the effect of SP exposures in the cervix is predominantly on inflammatory responses that are associated with mDC recruitment.

## Discussion

We set up a model of repeated vaginal exposures to HIV-1 SP in MVA-HIV-B vaccinated NHPs to demonstrate the effect of semen during HIV-1 prevention studies, such as vaccination and challenge studies.

Our study characterized the impact of SP on local immunity in the FRT. Here, we showed recruitment of both CD8^+^ T cells to the LNs draining the FRT and CD11c^+^ dendritic cells to all compartments of the FRT. In addition to altering the proportion of mucosal immune cells, SP exposures also affected antigen-specific cellular responses. Indeed, HIV-1^+^ SP exposures increased responses against vaccine vector mediated by CD4^+^ and CD8^+^ T cells. SP clearly influenced the activation of immune cells as there was an increase in CD154 marker expression in vaginal tissue exposed to SP, even before *in vitro* restimulation with MVA. Moreover, SP exposures showed anti-MVA responses mediated by CD4^+^ T-cells mainly in the cervix. This CD4^+^ T-cell response was not observed in unexposed mucosae.

The anti-MVA CD8^+^ T cell response was also affected by SP exposures, once again mainly in the cervix. Indeed, the frequency of anti-MVA CD8^+^ T cells producing IFN-γ, MIP-1β, TNF-α, or IL-2 increased after SP exposures. Antigen-specific CD8^+^ T cells were specifically recruited following SP exposures, as the percentage of total CD8^+^ T cells was not modified. Such recruitment could be due to the pro-inflammatory chemokines found in HIV-1^+^ SP (direct effect) (21) or factors produced by SP-stimulated FRT epithelial cells (indirect effect) (32). In addition to affecting the strength, SP exposures also affected the quality of the response mediated by anti-MVA CD8^+^ T cells in the FRT. Thus, MVA-specific CD8^+^ T cells producing three or four cytokines per cell were more frequent in the cervix after SP exposures. This increase may be related to the inflammatory environment induced by SP. Indeed, pro-inflammatory cytokines in HIV-1^+^ SP could attract cells carrying a co-stimulatory signal for CD8^+^ T cells. Co-stimulatory signals for T cells are classically provided by antigen presenting cells (33). HIV-1^+^ SP exposures induced specific recruitment of CD11c^+^ DC, which could affect MVA-specific CD8^+^ T cell stimulation. Our enrichment analysis of the transcriptional profile from cervical tissue showed that endocytosis-related pathways were downregulated by SP exposures, whereas the antigen presentation pathway was significantly upregulated. This pattern suggests a dendritic cell maturation profile, as mature dendritic cells have a lower endocytic capacity than immature dendritic cells associated with antigen presentation specialization (34). Thus, the association of pro-inflammatory cytokines of HIV-1^+^ SP and the recruitment of mature CD11c^+^ APC could be responsible for the higher quality of the CD8^+^ T cell response after SP exposure. However, APC recruited after HIV-1^+^ SP exposures could locally constitute new target cells for HIV-1. Moreover, antigen-specific CD4^+^ T cells detected after SP exposures could also constitute target cells for the virus. Thus, a challenge study following HIV-1^+^ SP exposures may help to determine whether the effects that we characterized increase protection against or susceptibility to HIV-1 infection.

Although we detected MVA-specific humoral responses in body fluids, we did not observe an effect of SP exposures on levels of anti-MVA antibodies in vaginal secretion. Further analyses to determine whether SP exposures affect the functions of secreted anti-MVA immunoglobulin, such as its neutralization capacity, may be informative.

Transcriptomic analyses showed that the expression of inflammation-related chemokine CCL26 and factors involved in protection against oxidative stress and inflammation, such as PRDX2 and metallothioneins (MTE, MT1X, and MT2A) significantly increased after SP exposures. Upregulation of these factors suggests that our analyses were performed after the peak of inflammation. Indeed, our analyses were performed 24 h after the last SP inoculation, and thus we may have missed the upregulation of early inflammatory genes such as COX-2 (10), and rapidly recruited immune cells, such as neutrophils (35). Our results are consistent with those of previous studies performed in women, which showed a local effect of HIV-1^neg^ SP on cervical immune cells and inflammation (10).

Previous *in vitro* studies reported an opposite effect of SP exposure on lymphocyte immunity (36, 37). Indeed, it has been shown that SP downregulated T cell and NK cell functions, such as cytotoxicity and cytokine production. The origins of the cells may explain this discrepancy with our results. The inhibitory effect of SP on T cells was observed after direct contact of SP with cells from peripheral blood (36). In the lower FRT, SP contact with T cells could be facilitated due to microtrauma of the epithelium during intercourse. However, the intraepithelial T cells are not abundant in the lower tract (38) so SP interacts mainly with epithelial cells (10). Thus, SP stimulates epithelial cells, which then produced soluble factors, inducing cell recruitment and T-cell activation (32, 39, 40). SP could also directly stimulate the DC found in FRT epithelium. *In vitro* studies have demonstrated that SP induces the production of soluble factors by epithelial cells, including pro-inflammatory cytokines (41). Among these, follistatin, also highlighted by our study is of great interest. Indeed, the function of this glycoprotein is to bind and inhibit members of the TGFβ family, in particular, activin (28). The loop of activin/follistatin process appeared in our transcriptional analysis on FRT as expression of the activin A receptor type A2 (ACVR2A gene) that was upregulated after SP exposures and correlated with CD11c^+^ mDC recruitment (Figures 5A,B). Furthermore, high concentrations of follistatin are found in SP (42) and SP regulates the synthesis of activin and follistatin (43). Moreover, follistatin has been shown to increase the function of antigen-specific CD8^+^ T cells (44). Future studies to investigate the role of follistatin in the upregulation of MVA specific CD8^+^ T cell function after SP exposures in cervical tissue may be informative.

In conclusion, our combined analysis of molecular and cellular events shows that HIV-1^+^ SP affects mainly on cervical immune cells and MVA-specific T cell responses. Further studies are required to characterize the role of each protagonist (*i.e*. factors in SP, epithelial cells, and HIV-1^+^ particles) in the increase of the strength and quality of vaccine-CD8^+^ T cell response after SP exposures.

## Ethics Statement

Twelve sexually mature adult female cynomolgus macaques (Macaca fascicularis) imported from Mauritius were housed in the Infectious Disease Models and Innovative Therapies (IDMIT) facilities at the Commissariat à l'Energie Atomique et aux Energies Alternatives (CEA, Fontenay-aux-Roses, France). The treatment of NHPs at the CEA complies with French national regulations (CEA authorization A 92-032-02), the Standards for Human Care and Use of Laboratory Animals (OLAW Assurance number #A5826-01), and European Directive 2010/63 (recommendation #9). Experiments were supervised by veterinarians in charge of the animal facility. This study was approved and accredited by the Ethics Committee on Animal Experimentation (Comité d'Ethique en Expérimentation Animale) of the CEA (A14-080) and the French Research Ministry. Animals were housed in pairs under controlled humidity, temperature and light (12-h light/dark cycles). Water was available *ad libitum*. The animals were monitored and fed once or twice a day with commercial monkey chow and fruits, by trained personnel, and were provided with environmental enrichment including toys, novel foodstuffs and music, under the supervision of the CEA Animal Welfare Officer.

## Author Contributions

RM, YL, FB-S, GS, RL, and EM: study conception and design. SN and ND-B: resources. RM, M-TN, MP, CL, HH, CC, and ND-B: acquisition of data. RM, NT, GS, RL, and EM: analysis and interpretation of data. RM, M-TN, NT, RL, and EM: drafting of manuscript. M-TN, NT, FB, YL, RL, and EM: critical revision.

### Conflict of Interest Statement

The authors declare that the research was conducted in the absence of any commercial or financial relationships that could be construed as a potential conflict of interest.
