# Supplementary material for: Seminal Plasma Exposures Strengthen Vaccine Responses in the Female Reproductive Tract Mucosae
Source: Front Immunol. 2019 Mar 12;10:430. doi: 10.3389/fimmu.2019.00430 (PMC6423065; doi:10.3389/fimmu.2019.00430)
Supplement: Supplementary file 1 [file Data_Sheet_1.PDF]

Seminal plasma exposures strengthen vaccine responses in the female reproductive tract mucosae

Romain Marlin[1,2,3], Marie-Thérèse Nugeyre[1,2,3], Nicolas Tchitchek[1], Matteo Parenti[1,3], Cécile Lefebvre[3,4], Hakim Hocini[3,4], Fahd Benjelloun[1,2], Claude Cannou[1,2], Silvia Nozza[5], Nathalie Dereuddre-Bosquet[1], Yves Levy[3,4,6], Françoise Barré-Sinoussi[3,7], Gabriella Scarlatti[3,8], Roger Le Grand[1,3], and Elisabeth Menu[1,2,3]

1-CEA, Université Paris-Sud, Inserm, U1184 « Immunology of viral infections and autoimmune diseases » (IMVA), IDMIT Department, IBFJ, Fontenay-Aux-Roses, France

2-MISTIC group, department of Virology, Institut Pasteur, Paris, France

3-Vaccine Research Institute – VRI, Hôpital Henri Mondor, Créteil, France

4-Institut Mondor de Recherche Biomédicale - INSERM U955, équipe 16 physiopathologie et immunothérapies dans l'infection VIH, Créteil, France

5-Infectious Diseases Department, San Raffaele Scientific Institute, Milan, Italy

6-Assistance Publique-Hôpitaux de Paris (AP-HP), Groupe Henri-Mondor Albert-Chenevier, Service d'immunologie clinique, Créteil, France

7-International division, Institut Pasteur, Paris, France

8-Viral Evolution and Transmission Unit, San Raffaele Scientific Institute, Milan, Italy

Correspondence: Dr Elisabeth Menu; elisabeth.menu@pasteur.fr

Table S1. Antibodies used in immune phenotyping experiments.

**Antibody**	**Fluorochrome**	**Clone**	**Supplier**
**CD45**	PerCp	D058-1283	BD pharmingen
**CD3**	V500	SP34-2	BD Horizon
**CD4**	V450	L200	BD Horizon
**CD8**	BV650	RPA-T8	BD Horizon
**CD20**	PE-CF594	2H7	BD horizon
**HLA-DR**	APC-H7	G46-6	BD pharmingen
**CD20**	BV711	2H7	BD horizon
**NKG2A**	PE	Z199	Beckman Coulter
**CD11c**	APC	S-HCL-3	BD
**CD14**	Alexa-700	M5E2	BD pharmingen
**CD66**	FITC	TET2	Miltenyi
**CD123**	PC7	7G3	BD pharmingen

Table S2. Antibodies used in ICS experiments.

Antibody	Fluorochrome	Clone	Supplier
**CD3**	APC-Cy7	SP34-2	BD pharmingen
**CD45**	PerCP	D058-1283	BD pharmingen
**CD8**	V500	RPA-T8	BD pharmingen
**CD154**	FITC	TRAP1	BD pharmingen
**IL2**	APC	MQ1-17H12	BD pharmingen
**IFN-γ**	V450	B27	BD horizon
**TNF-α**	A700	BMAb11	BD pharmingen
**MIP-1β**	PE	D21-1351	BD pharmingen
**CD4**	PC7	L200	BD pharmingen

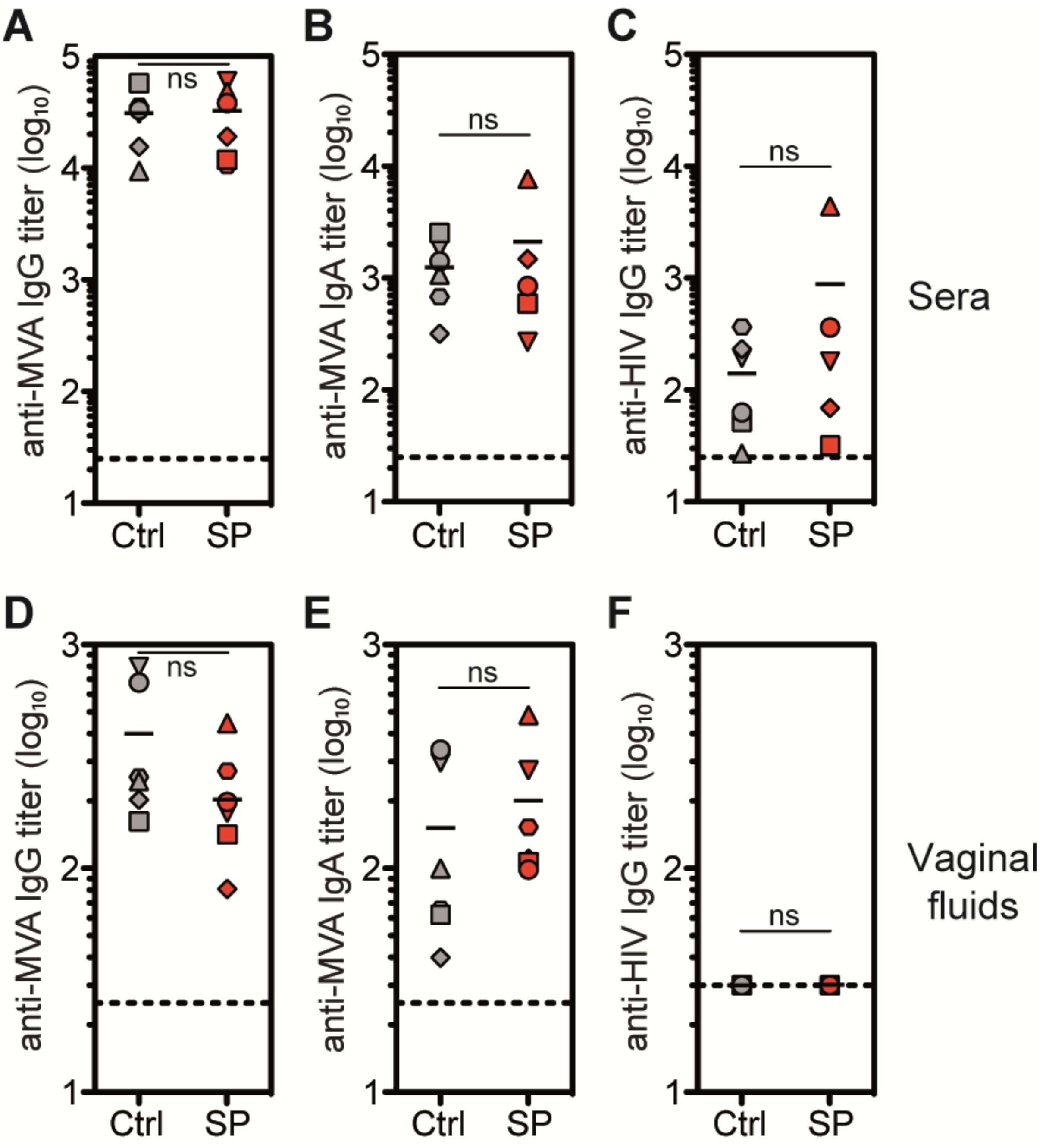

Figure S1. SP exposures do not alter vaccine humoral responses

Titers of MVA specific IgG (A, D) and IgA (B, E) were determined in sera (A, B) and vaginal fluids (D, E), the day after the last intravaginal SP exposure. Titers of HIV-1 (p24)-specific IgG were also measured in sera (C) and vaginal fluids (F). Control group animals (Ctrl) are indicated in grey and SP group animals (SP) in red. Each symbol represents one animal.

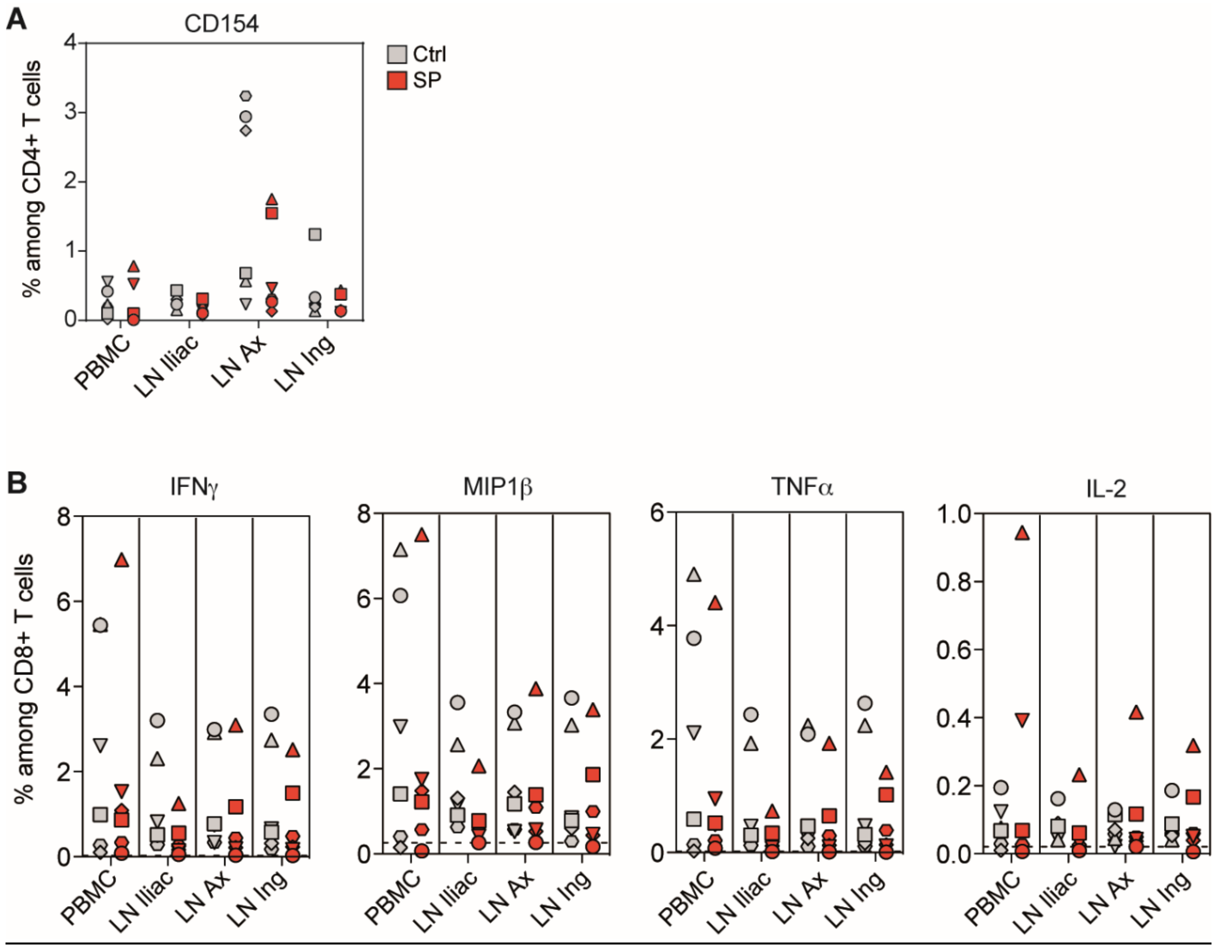

<u>Figure S2. MVA-specific T cell responses in the blood and LNs.</u>

(A) Percentage of $CD154^{+}$ cells among MVA-specific $CD4^{+}$ T cells in the blood and LNs after *in vitro* stimulation. (B) Percentage of IFN-γ^{+}, MIP-1β^{+}, TNF-α^{+}, and IL-2^{+} cells among MVA-specific $CD8^{+}$ T cells in the blood and LNs after *in vitro* stimulation. Control animals (Ctrl) are indicated in grey and SP exposed animals (SP) in red.

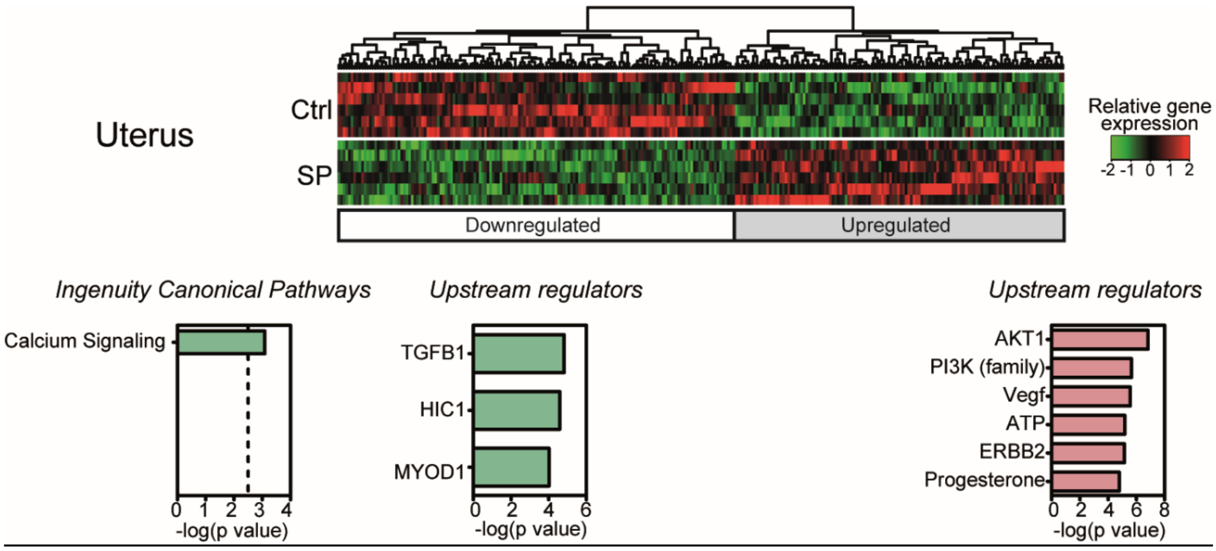

Figure S3. Impact of SP exposures on associated canonical pathways and upstream regulators for DEG in uterine tissue.

Heatmaps of relative gene expression in the uterus. Associated canonical pathways and upstream regulators for down-regulated (green) and up-regulated (red) genes after SP exposures are detailed.
